# Depression and Health Related Quality of Life in Adolescent Survivors of a Traumatic Brain Injury: A Pilot Study

**DOI:** 10.1371/journal.pone.0101842

**Published:** 2014-07-10

**Authors:** Ashley Di Battista, Celia Godfrey, Cheryl Soo, Cathy Catroppa, Vicki Anderson

**Affiliations:** 1 School of Behavioural Science, University of Melbourne, Melbourne, Australia; 2 Department of Psychology, The Hospital for Sick Children, Toronto, Ontario, Canada; 3 Clinical Sciences, Murdoch Children's Research Institute, Royal Children's Hospital, Melbourne, Australia; 4 Psychology, Royal Children's Hospital, Melbourne, Australia; 5 Department of Paediatrics, University of Melbourne, Melbourne, Australia; University of Missouri-Kansas City, United States of America

## Abstract

Traumatic brain injury is (TBI) a leading cause of morbidity and mortality in youth. Adult survivors of a severe pediatric TBI are vulnerable to global impairments, including greater employment difficulties, poor quality of life (HRQoL) and increased risk of mental health problems. When estimating the health related quality of life in adolescents, the presence of anxiety and depression and the quality of social relationships are important considerations, because adolescents are entrenched in social development during this phase of maturation. The influence of anxiety, depression and loneliness on health related quality of life in adolescent survivors of TBI has not been documented. This pilot study aimed to identify and measure the relationship between anxiety, depression and loneliness and perceived health related quality of life in adolescent survivors of a TBI. Method: mixed method/cohort pilot study (11 adolescents, mild to severe TBI; 9 parents), using self-report and proxy-report measures of anxiety, depression, health related quality of life, loneliness and clinical psychiatric interviews (adolescent only). Results: Self-reported depression was significantly correlated with self-reported HRQoL (*rs* [11] = −0.88, *p*<0.001). Age at injury was significantly correlated with self-reported HRQoL (*rs* [11] = −0.68, *p* = 0.02). Self-reported depression predicted self-reported HRQoL (R^2^ = 0.79, F [1, 10] = 33.48, *p*<0.001), but age at injury did not (R^2^ = 0.19, F [1, 10] = 2.09, *p* = 0.18). Conclusions: Our results suggest that depression is a predictor of health related quality of life in youth post-TBI. The possibility of using targeted assessment and therapy for depression post-TBI to improve health related quality of life should be explored.

## Introduction

Traumatic brain injury (TBI) is a leading cause of morbidity and mortality in children and adolescents in first world nations [1]. In recent years there has been a move towards assessing sequelae of TBI beyond cognitive domains, including quality of life (HRQoL) and mood disorders, such as ADHD, depression and anxiety post-injury [2]. Research from our team investigating adult survivors of pediatric TBI has reported that survivors of severe TBI are particularly vulnerable to global functional impairments, including poorer school performance, greater employment difficulties, poor HRQoL and increased risk of mental health problems [3]. However, the majority of research into pediatric and adolescent TBI outcomes in the psychosocial domain focuses on parent or clinician proxy assessment. The appropriateness of proxy reporting for internalizing conditions, such as quality of life (QoL), depression and anxiety has been criticized for many years in the broader psychology literature [4], [5], yet parental proxy reporting remains the most often used method of assessment for these states in the pediatric TBI field [6].

### Epidemiology: Anxiety, Depression and HRQoL

Anxiety disorders are the most commonly diagnosed mental disorders in childhood and adolescence [7]. There is a high point prevalence of depression in otherwise healthy adolescents, with estimates as high as 6% [8]. In addition, there is a strong co morbidity between depression and anxiety, with reported co morbidity as high as 90% in those with an already diagnosed anxiety disorder experiencing a concurrent depressive episode [9].

The recent systematic review of HRQoL in pediatric survivors of a TBI [6] highlighted that all of the data available on pediatric HRQoL post-TBI are dependent on proxy reporting (clinician or physician), and adhere to the HRQoL paradigm, most frequently employing the Pediatric Quality of Life Inventory (PedsQL 4.0; [10]). Our systematic review [6]also found that good outcomes were contingent on milder injuries, proxy reporting and early assessment whereas poor outcomes occurred in the context of more severe injuries and later assessment (≤6 months vs. ≥1 year post-trauma, respectively). Recent work from our group has identified that the relationship between parental report and self-report in adolescent HRQoL ratings is poor and caution needs to be taken when interpreting HRQoL data derived from solely parent proxy sources [11].

### Current Research in Adolescent Anxiety, Depression and HRQoL

The small body of literature on affective symptomatology and disorders following pediatric TBI has begun to describe elevated levels of anxiety and depression following brain injury in both children and adolescents [12]–[16]. These data, however, are plagued by methodological constraints, most notably the use of parent-proxy observers to rate anxiety and depression symptoms [4], [5]. The few studies that have used diagnostic interview or self-report scales suggest a link between TBI, anxiety and depression [2], [14]–[17]. Recent data [2] have also identified the development of novel definite or subclinical anxiety disorders in children during the first six months after a TBI, but no information is available on later time points (e.g. beyond the relatively acute post-injury period of 6 months), or for older adolescents. The limited data are consistent with adult TBI literature which shows linkages between brain injury and the development of new onset disorders or persistence and worsening of pre-existing anxious or depressive conditions [18], [19].

### Methodological Constraints – Concordance between Self-Report and Parent Proxies on Measures of Anxiety, Depression and HRQoL

The concordance between self-report and proxy-reporting of anxiety and depressive symptomatology reflects similar findings to those reported in the HRQoL literature. A meta-analysis of 119 studies by Acenbach, McConaughy and Howell [20] assessed the consistency between ratings of behavioral and emotional problems from various proxies, including parents, teachers, mental health workers, observers and peers and their child/adolescent counterpart. Overall correlations were higher for younger children with proxy reporting, but decreased with adolescents. The authors suggested that, given the overall modest correlations between proxies and children and adolescents, the process of using proxy reports are ineffective and promoted use of multiple sources to achieve the best possible ratings [20]. Kazdin, Esveldt-Dawson, Unis & Rancurello [21] have also reported little or no relationship between mother or father proxy reports and that of their children on measures of depression.

### Social Relationships in Adolescence – Impact on Anxiety, Depression and HRQoL

When estimating HRQoL and internalizing behaviours in adolescents, the quality of social relationships and friendships is an important consideration because adolescence is a period of intense and rapid social development. Adolescents are particularly sensitive to social comparison and concerns regarding their status among peers [22], [23]. Depression, anxiety and low self-esteem have been associated with peer difficulties during childhood and adolescence [24], [25]. Anxiety in children and adolescence has been linked to peer rejection [26]. The impact of loneliness on the adolescent post-TBI may be even more problematic when young people experience social withdrawal due to cognitive difficulties (e.g. remedial classes), social interaction problems (e.g. behavioural sequelae post-trauma) or functional impairments that limit interaction with others at school and leisure (e.g. motor co-ordination problems, speech impairment, etc). The compounding effects of cognitive, behavioural and social difficulties in adolescents post TBI [26], [27] make this group especially vulnerable to anxiety and depression and predictors of these affective conditions warrant investigation.

The aim of this study was to explore the role of anxiety, depression and loneliness and their association with perceived HRQoL in adolescent survivors of a TBI. The concordance between parent proxy and adolescent self-report on measures of anxiety, depression and HRQOL was also explored.

We hypothesized that: 1.Self-reported anxiety and or depression would be related to poorer self-reported HRQoL; 2. that loneliness would be associated with greater depression and anxiety, as well as poorer HRQoL ratings from adolescents; 3.there would be poor concordance between all proxy and self-report measurements on the self-reported and parent proxy reported anxiety, depression and QoL measures.

## Methods

### Ethics Statement

The study was approved by the Royal Children’s Hospital (RCH) Human Research Ethics Committee on 11 January, 2011. HREC 30198 A, Quality of life in adolescents following traumatic brain injury: the impact of anxiety and depression. Date of original approval: 11 January 2011. Duration: 36 months. Date of approval expiry: 11 January 2014. Please note that this application was recommended for Chairman’s approval (expedited review). All Chair approvals are ratified at the subsequent Human Research Ethics Committee (HREC) meeting. In the interim, the HREC require the approved materials to be used, as listed on the attached Approval Certificate. The Royal Children’s Hospital Human Research Ethics Committee (RCH HREC) is constituted in according to the National Health and Medical Research Council’s ‘National Statement on Ethical Conduct in Human Research (2007). The committee operates in accordance with these guidelines and is registered with the NHMRC.

All participants were required to provide written consent to participate in the study, in the form of a signed consent letter (parents and/or legal guardians) and assent forms for adolescents. All participants in this study provided written informed consent from parents or guardians on behalf of the minors/children enrolled in this study.

### Procedure

Potential participants were identified via: 1. clinical audits of admission to the Emergency Department; 2. private referrals; 3. participants previously enrolled in other studies who agreed to future contact about upcoming studies conducted at RCH.

Assessments were conducted at the RCH in outpatient clinics, in a private room. Parents were asked to complete parent versions of questionnaires while they were waiting for the young people to complete the assessment. For older participants who did not attend with a parent (e.g. 18 years and older) questionnaires were supplied to the adolescent to give to their parent. Completion of parental questionnaires was not mandatory for participants aged 18 and over. For those families who agreed but could not attend RCH for the assessment (n = 1), questionnaire packages were mailed to the home, along with consent forms to sign and return (with a postage paid return envelope provided). Rural participants who could not attend RCH (n = 1) were also offered the opportunity to conduct the clinical interview (K-SADS, SCID; see measures section) over the telephone.

### Participants

**Figure 1:**
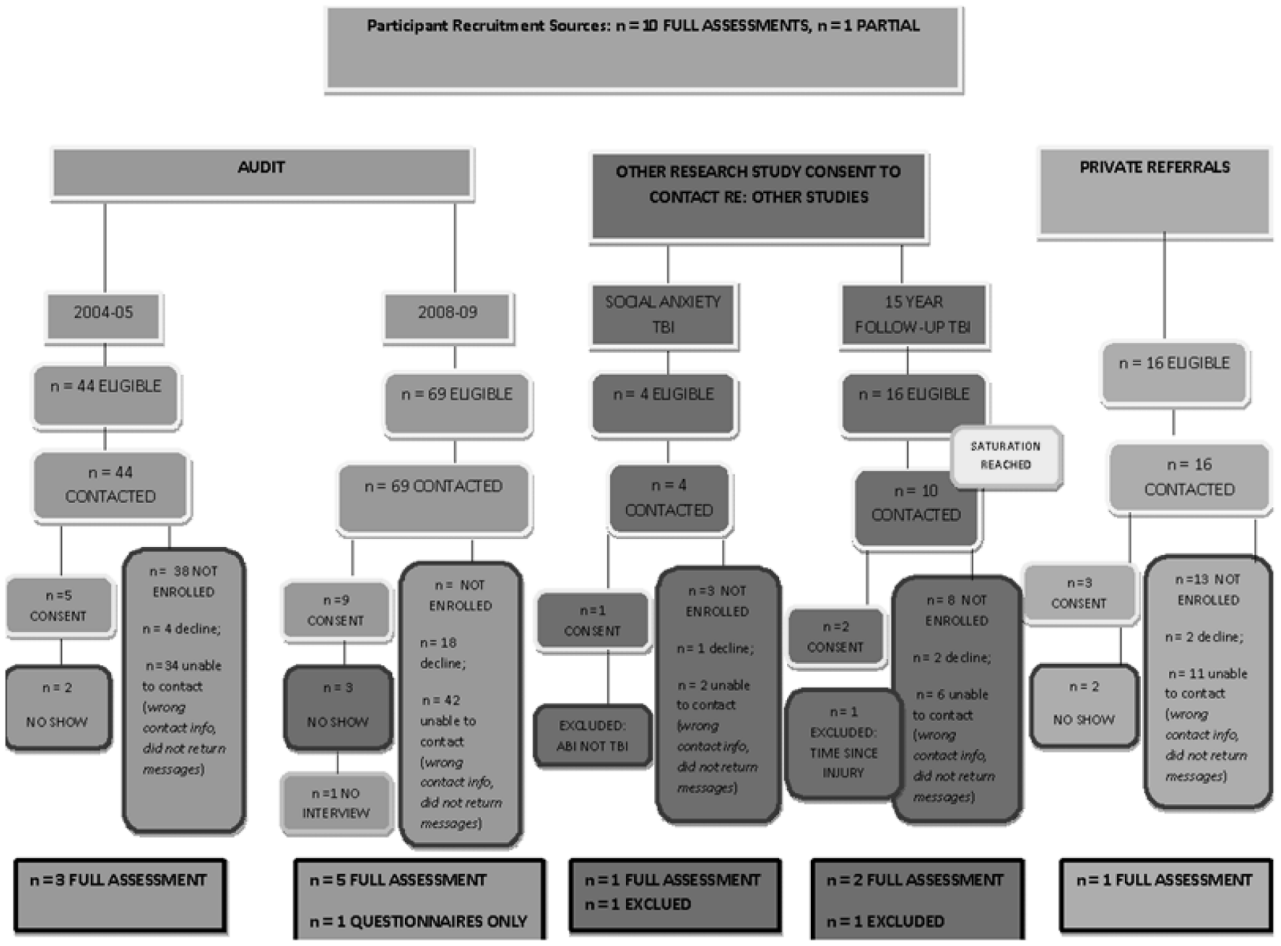
Participant Recruitment and Final Sample Flow Diagram. This figure documents the participant recruitment process, sources, participation and decline rates, accounting for the final sample.

A total of 581 patients were identified via two clinical audits and private referrals (see Figure 1). Correcting for duplicate and non-TBI entries, a total of 153 were deemed eligible to contact, based on the inclusion criteria. In accordance with ethics approval and associated Australian privacy laws, a tracing letter and follow up phone call were provided to all 153 families. Two families were excluded due to difficulties with English language identified via phone call. A total of 106 potential participants could not be contacted (e.g. outdated phone number, outdated address). Of the remaining 47 eligible families, 27 families declined participation. No reasons were provided. Twenty families consented to participate. Of these, 7 did not attend. Two participants were fully assessed but later excluded from analyses, due to etiological and methodological issues. One participant was excluded after assessment due to etiology of trauma (acquired brain injury (ABI) via tumor, not TBI; incorrect documentation) and one participant was excluded due to the time since injury, which was double that of the other participants (16 years post trauma) and represented a significant outlier in terms of time since injury. As a result, a total of 11 full cases were analyzed. Nine parents participated in the assessment, rendering a total final sample of n = 20. Attrition analyses revealed no significant differences between participants and non-participants on TBI severity *X* ^2^ (1, N = 152) = 1.60, *p* = 0.21, age at injury *X* ^2^(4, N = 152) = 2.27, *p* = 0.69 or gender *X* ^2^ (1, N = 152) = 0.52, p = 0.47.

### Inclusion/Exclusion Criteria

Inclusion criteria were: 1). Aged 10–25 years at time of approach and assessment; 2). Diagnosis of TBI, 3). Medical records sufficient to determine injury severity; 4). No pre-injury history of neurological, developmental, or psychiatric disorder; 5). English speaking; 6.) minimum of one year post TBI. Exclusion criteria were: non-English speaking, non-accidental injury, and pre-injury diagnosis of neurological, developmental, or psychiatric disorder, IQ below 70.

### Measures

#### 1. DEMOGRAPHICS AND INJURY CHARACTERISTICS

Socioeconomic status, age, gender, IQ, age at injury, injury severity and time since injury were collected. IQ was assessed using the two-subtest form of the Wechsler Abbreviated Scale of Intelligence (WASI; [28]). The two-subtest form yields a full scale IQ (FSIQ). Standardized age appropriate norms were recorded. As a result of inconsistent injury severity data recorded in patient medical files, the TBI severity was classified according to the Mayo Classification System for Traumatic Brain Injuries [29]. The Mayo Classification System [29] was used as it was designed to permit TBI severity classifications of injuries in instances where data relating to the injury, e.g. post-traumatic amnesia duration (PTA), loss of consciousness (LOC) duration, etc., may be missing. The Mayo Classification System maximally uses the available information to classify TBIs into the following categories: (a) Moderate-Severe (Definite) TBI, (b) Mild (Probable) TBI, (c) Symptomatic (Possible) TBI.

#### 2. QUESTIONNAIRES

The questionnaires were completed in order to screen for current, point-prevalence (e.g. most recent seven days) of anxiety and depressive symptomatology. Cut off scores (where available) and total scores were used to identify experiences of both anxiety and depression. Individual adolescents were administered all of the self-report measures plus a clinical interview, using the age-appropriate version. Parent proxies completed the CDI, SCARED and PedsQL parent proxy reports.

- **ANXIETY:** Participants were administered either the Screen for Anxiety Related Disorders (SCARED [30]; ≤18 years old) or the State-Trait Anxiety Inventory (STAI [31]; 19–25 years)
  - The SCARED was administered to those participants who were ≤18 years old. The SCARED is a 41-item self-report questionnaire assessing five domains of anxiety: Generalized Anxiety Disorder, Separation Anxiety Disorder, Social Anxiety Disorder, Significant School Avoidance and Panic Disorder/Significant Somatic Symptoms. A total score is also provided, where scores ≥25 indicate the presence of an anxiety disorder, and those with scores ≥30 are more specific of a disorder. Cut off scores for the SCARED are supplied for a “Total Anxiety” score as well as five subtest: Panic/somatic, general anxiety, separation anxiety, social phobia and school phobia. Cut off scores are those provided by SCARED developers, who generated cut off values for optimal sensitivity and specificity.
  - The STAI was administered to those participants who were aged 19 years and older. The State-Trait Anxiety Inventory Form Y (STAI) clearly differentiates between the temporary condition of “state anxiety” and the more general and long-standing quality of “trait anxiety.” The STAI-assesses feelings of apprehension, tension, nervousness, and worry. Individuals respond to each item on a four-point Likert scale, indicating the frequency with which each strategy is used.
- **DEPRESSION**− the Child Depression Inventory (CDI [32]; ≤17 years) or the Centre for Epidemiology Studies Depression Scale (CESD [33] 18–25 years).
  - the Child Depression Inventory (CDI) was administered to those participants aged ≤17 years). The CDI is a 27 item self-report questionnaire assessing feelings and thoughts related to depression in the past 2 weeks. Each item consists of three statements that are ranked on a Likert-type scale from 0–2 for severity. Total scores range from 0–54. There are five subscales to the CDI, including: “Negative mood”; “Interpersonal Problems”; “Ineffectiveness”; “Anhedonia”; “Negative Self-Esteem”. Cut-off values of raw scores and t-scores are available for the self-report versions of the CDI, but not for the parental proxy. Raw scores ≥19 (t-score ≥65) endorse a clinically significant level of depression.
  - the Centre for Epidemiologic Studies Depression Inventory (CES-D) was administered to those participants aged >17 years. The scale contains 20 questions, and each item is rated on a scale from 0 to 3 on the basis of “how often you have felt this way during the past week”: 0 = rarely or none of the time (less than 1 day), 1 = some or a little of the time (1–2 days), 2 = occasionally or a moderate amount of time (3–4 days), and 4 = most or all of the time (5–7 days). Total severity is calculated by summing all of the scores. Scores range from 0 to 60; higher scores indicate more severe depressive symptoms. A cut-off score of 16 is indicative of “significant” or “mild” depressive symptomatology.
- **LONELINESS:** Loneliness was assessed using the Peer network and Dyadic Loneliness Scale (PNDLS [34] for adolescents up to 17 years of age, and The Differential Loneliness Scale for Non-Student Populations [35] were used for those adolescents aged 18 and older.
  - The Peer Network and Dyadic Loneliness Scale (PNDLS) was administered to those adolescents 17 years of age and younger. The PDNLS is a 16 item, four point scale self-report measure. The PNDLS yields two subscale scores, one for peer network loneliness and one for peer dyadic loneliness. Higher scores indicate greater loneliness. Scores are computed for each subscale by summing the child’s self-ratings on the eight items comprising the subscale and dividing by eight. Therefore, subscale scores range from 1 (very low loneliness) to 4 (very high loneliness).
  - The Differential Loneliness Scale for Non-Student Populations assesses loneliness in the context of: familial relationships, romantic relationships, friendships, relationships with family and with larger groups. The self-report measure contains 60 true/false questions and was administered to those participants aged 18 and older.
- **QUALITY OF LIFE:** Participants (*aged <19 years of* age) and their parents were given the PedsQL 4.0 [10], a self-report measure to assess current quality of life, or the SF-36 version 2 [36] for participants aged 19–20 years.
  - The PedsQL 4.0 is a 20 item self-report questionnaire that assesses five domains of quality of life: 1.) physical functioning (8 items); 2.) emotional functioning (5 items); 3.) social functioning (5 items); and 4.) school functioning (5 items). Individual scales can be combined to yield 3 summary measures of physical (same as physical functioning scale), psychosocial (emotional, social and school functioning scales) and total health (all 4 scales). Scale scores range from 0 to 100; higher scores connote better quality of life.
  - The SF-36 is a self-report questionnaire that yields 8 scales (and two summary measures), assessing: 1.) physical functioning; 2.) physical role; 3.) bodily pain; 4.) general health; 5.) vitality; 6.) social functioning; 7.) emotional role; and 8.) mental health. The two summary indices separate the physical from the mental component of the health-related HRQoL. In norm-based scoring, each scale was scored to have same average (50) and the same standard deviation (10 points).
- **DIAGNOSTIC INTERVIEWS:** In addition to self-report measures, clinical interviews were employed to determine lifetime anxiety and or depression. The Kiddie-SADs-Present and Lifetime version (KSADS-PL [37]) was used for those aged 18 and younger and The Structured Clinical Interview for DSM-IV-TR, Research Non-Patient Edition (SCID-R: [38] First, Psitzer, Gibbon, Williams, 1997) was used for participants over the age of 18 years. The original scoring method provided by the developers of the SCID and the KSADS-PL were employed.
  - The Kiddie-Sads-Present and Lifetime version (KSADS-PL) primary diagnoses assessed with the K-SADS-PL include: Major Depression, Dysthymia, Mania, Hypomania, Cyclothymia, Bipolar Disorders, Schizoaffective Disorders, Schizophrenia, Schizophreniform Disorder, Brief Reactive Psychosis, Panic Disorder, Agoraphobia, Separation Anxiety Disorder, Avoidant Disorder of Childhood and Adolescence, Simple Phobia, Social Phobia, Overanxious Disorder, Generalized Anxiety, Obsessive Compulsive Disorder, Attention Deficit Hyperactivity Disorder, Conduct Disorder, Oppositional Defiant Disorder, Enuresis, Encopresis, Anorexia Nervosa, Bulimia, Transient Tic Disorder, Tourette’s Disorder, Chronic Motor or Vocal Tic Disorder, Alcohol Abuse, Substance Abuse, Post-Traumatic Stress Disorder, and Adjustment Disorders. Only the modules pertaining to Mood Disorder (Depression and Suicidality) and Anxiety (Panic Disorder, Agoraphobia, Separation Anxiety Disorder, Avoidant Disorder of Childhood and Adolescence, Simple Phobia, Social Phobia, Overanxious Disorder, Generalized Anxiety, Obsessive Compulsive Disorder) were used in the assessment.
  - The Structured Clinical Interview for DSM-IV-TR, Research Non-Patient Edition (SCID-R) is for use in studies in which the subjects are not identified as psychiatric patients (e.g., community surveys, family studies, research in primary care). The diagnostic modules of the SCID-I/NP are the same as those of the SCID-I/P (W/PSYCHOTIC SCREEN); the only difference in the two versions is in the Overview section. In the SCID-I/NP there is no assumption of a chief complaint, and other questions are used to inquire about a history of psychopathology.

Diagnostic interviews were conducted by the lead author, who is a practicing psychologist and holds a Ph.D. in psychology. Participants were coded into de-identified study numbers before interview. The interviewer did not review the details of the case prior to interview, however, the interviewer was not blind to TBI severity or time since injury.

### Statistical Analysis

Data acquired were normal, albeit derived from a small sample. Given that the sample size was small non-parametric correlations were used. Correlation analysis of the relationship between self-reported anxiety and or depression on quality of life was conducted using Spearman Rank Correlations. Gender, social economic status, TBI severity, age at injury, time since injury and cognitive functioning were correlated with anxiety, depression, loneliness and HRQoL variables. Concordance between proxy and self reports was conducted via Spearman Rank correlation. Single factor linear regressions were performed using those independent variables which had strong correlations and effect sizes with the dependant variable (HRQoL) (see Results section). This process of using multiple single independent variable regressions was conducted in order to account for the small sample size (e.g. inefficient power for multiple variable entries) and to determine directionality of relationship versus simple correlations.

As different measures were required for different aged participants, the measures specific to the domain of interest were combined to create grouped variables. In order to ensure that all measures were assigned a value appropriate to a standard metric, all total raw scores were re-coded using the lowest common denominator (LCD). The LCD was calculated and all variables were multiplied by their corresponding value to render each appropriate in the new metric. For example, if measure A was out of 12 and measure B was out of 20 would have resulted in the following calculation: LCD = 60; therefore [(Measure A Total Raw Score * 5)+(Measure B Total Raw Score *3)] = Combined A+B Measure. The measures were equivalent in terms of domains assessed, thus rendering the measures appropriate to be analyzed together. This process included the following: the SCARED and the STAI were combined to generate a total anxiety measure; the Peds QL was combined with the SF-36, the CDI’s (version 1 and 2) and the CESD were combined; the PDNLS and The Differential Loneliness scales were combined.

Frequency of endorsed symptomatology for clinically relevant anxiety and depression (parent proxy and self-reported) are presented in table 1. Parent proxy and self-reported ‘good’ versus ‘poor’ quality of life as well as endorsed loneliness rates are also presented in table 1. Life-time history of depression and anxiety are also reported. Life-time history of depression and anxiety are the subject of another companion paper, and so are reported briefly here.

**Table 1:** Anxiety, Depression and HRQoL Ratings Stratified by Self-Report and Parental Proxy Report.

Sample	Anxiety Clinically Relevant n, (%)	Depression Clinically Relevant n, (%)	Good Overall HRQoL n, (%)	Poor Overall HRQoL n, (%)	Peer Network Loneliness – Lonely n, (%)	Peer Dyadic Loneliness – Lonely n, (%)	Differential Loneliness – Lonely n, (%)
**Self-Report, Current Screen**	3, (27.2%)	2, (18.2%)	7, (67%)	4, (36.4%)	1, (9.1%)	4, (36.4%)	0, (0%)
**Parent Proxy**	1, (11.1%)	1, (11.1%)	7 (78.8%)	2, (22.2%)	N/A	N/A	N/A

## Results

Participant characteristics and injury details for n = 11 participants included in the final analyses are presented in Table 2. Information on TBIs in the group varied, with inconsistent information available across subjects (e.g. missing information on Glasgow Coma Score at scene, no documentation regarding Post-Traumatic Amnesia, etc). Using the Mayo Classification System for Traumatic Brain Injuries, the majority (63.6%) were moderate-severe definite. There were 6 moderate-severe (definite), 3 mild (probable) TBI and 2 symptomatic (possible) TBI in the final analysis. The average age at injury was 12.48 (3.06) years [range: 4.33–16 years]. The average age at assessment was 17.09 (1.81) years [range: 13.92–19.5 years]. The average time since injury at assessment was 4.62 (2.89) years [range: 1.92–10.75 years].

**Table 2:** Participant Demographics and Injury Characteristics.

GENDER	AGE INJURY,*AGE AT* *ASSESSMENT*	TIME SINCEINJURY	CAUSE OF INJURY	TYPE OF INJURY	GCS Scene;*GCS Lowest*	PTA(days,hours)	LOC	SURGICALINTERVENTION(YES, NO)	CT (Abnormal,Normal)	NEUROLOGICALSIGNS(present, absent)	TBISEVERITY
M	16 y,*17 y 9 m*	1 yr 11 mo	MVA (*occupant*)	Acceleration/deceleration	NA; GCS = *4*	21 DAYS	NA	NO	ABNORMAL	PRESENT	MSD
F	11 y 9 m,*13 y, 11 M*	2 yrs 2 mo	Sports-related headcollision withstationary object	Direct Impact, headagainst object.Skull fracture.	NA; *GCS = 12*	24 HOURS	<1 MIN	YES	ABNORMAL	PRESENT	MSD
M	14 y, 10 m*17 y, 1 m*	2 yrs 3 mo	Sports-related headcollision with ground	Direct impact, headagainst object	NA; *GCS = 13*	NA	<1 MIN	NO	NORMAL	PRESENT	MP
M	13 y, 3 m*15 y, 5 m*	2 yrs 2 mo	Sports-related headcollision with ground	Direct impact, headagainst object	NA; *NA*	NA	SHORTDURATION	NO	NORMAL	PRESENT	MP
M	12 yrs,10 mo *17 y*	4 yrs 2 mo	MVA (*occupant*)	Acceleration/deceleration	NA; *GCS = 3*	NA	NA	YES	ABNORMAL	PRESENT	MSD
*F	15 yrs, 0 mo*18 y, 5 m*	3 yrs 5 mo	Violence/assault	Direct impact, blowto the head	NA; *NA*	NA	NA	NO	NORMAL	PRESENT	SP
M	12 yrs, 3 mo*19 y, 2 m*	6 yrs 11 mo	Violence/assault	Direct impact, headagainst object	NA; *NA*	NA	NA	NO	NORMAL	PRESENT	SP
M	4 yrs, 4 mo*15 y, 1 m*	10 yrs 9 mo	MVA (*occupant*)	Acceleration/deceleration	NA; *GCS = 7*	NA	NA	YES	ABNORMAL	PRESENT	MSD
F	11 yrs, 3 mo*18 y, 6 m*	7 yrs, 3 mo	MVA (*occupant*)	Acceleration/deceleration	NA; *NA*	NA	SHORTDURATION	NO	ABNORMAL	PRESENT	MSD
F	12 yrs, 9 mo*19 y, 6 m*	6 yrs, 7 mo	MVA (*pedestrian*)	Direct impact, headagainst object	NA; *GCS = 13*	NA	NA	NO	ABNORMAL	PRESENT	MSD
M	13 yrs, 0 mo*16 y*	3 yrs, 0 mo	Sports-related headcollision with ground	Direct impact, headagainst object	NA; *NA*	NA	SHORTDURATION	NO	NORMAL	PRESENT	MP

NA = information was not documented in medical file; Short Duration = written as “short duration” in the medical file, no time/quantifiable duration recorded; TBI severity reported according to Mayo Classification System [29]; SP = symptomatic possible TBI; MP = mild probable TBI; MSD = Moderate-severe definite TBI. *Previous skull fracture as infant (<1 year of age).

The majority of the sample (64%) was male. The majority of the sample (54.5%) was enrolled in high school at the time of assessment. Three (27.3%) of the participants were enrolled in university studies, one (9.1%) was enrolled in a apprenticeship course, and one (9.1%) was employed in full-time work. The socio-economic status of the sample (Australian Bureau of Statistics Socio-Economic Indices for Areas; SEIFA 2006 [39] ) ranged from the third decile (e.g. lowest 30% of population) with a relative socio-economic disadvantage decile of 6 to the highest possible status (decile = 10; relative socio-economic disadvantage = 10). One participant fell below the 6^th^ decile. The majority of participants (91%) were at or above the 6^th^ decile, with an average decile of 7.8, The FSIQ was available for nine participants; two did not complete the assessment, one as a result of completing questionnaire packages and returning them via mail (rural participant) and the other was unable to complete the assessment in the time available. Of the nine participants assessed, the average FSIQ was 104 (15.3), range: 83.3–133.0.

Two of the participants did not have corresponding parental data; all analyses correlating parent and proxy data are based on the n = 9 full data sets.

Lifetime presence of clinically significant anxiety and or depression was assessed in 10 participants (one declined interview). Lifetime anxiety was present in 2 participants, (aged 15 years and 12 years, 3 months respectively at the time of injury). Lifetime depression was present in one participant. The one participant who endorsed previous lifetime depression also experienced co-morbid anxiety. Table 1 outlines the lifetime anxiety and depression reported, via interview, as well as the endorsed self-reported and parent proxy reported anxiety and depression data.

None of the parental proxy and self-report measures were correlated: HRQoL (*rs* [9] = −0.27, *p* = 0.49); anxiety (*rs* [9] = 0.65, *p = *0.06); depression (*rs* = 0.07, *p* = 0.86).

Self-reported depression was significantly correlated with self-reported HRQoL (*rs* [11] = −0.88, *p*<0.001). Loneliness was significantly correlated with anxiety (*rs* [11] = 0.72, *p* = 0.01) but not depression (*rs* [11] = −0.43, *p* = 0.19) or HRQoL (*rs* [11] = 0.37, *p* = 0.27). Self-reported anxiety was not correlated with self-reported HRQoL (*rs* [11] = −0.02, *p* = 0.95). FSIQ was not correlated with any of the outcome variables: QoL (*rs* [9] = −0.49, *p* = 0.19), depression (*rs* [9] = −0.63, *p* = 0.07), anxiety (*rs* [9] = −0.10, *p* = 0.81) or loneliness (*rs* [9] = −0.0, *p* = 0.99).

Age at injury was significantly correlated with self-reported HRQoL (*rs* [11] = −0.68, *p* = 0.02). Age at testing was significantly correlated with self-reported anxiety (*rs* [11] = −0.66, *p* = 0.03). Injury severity was not correlated with any of the self-reported outcome variables: anxiety (*rs* [11] = 0.44, *p* = 0.18), depression (*rs* [11] = 0.29, *p* = 0.39), HRQoL (*rs* [11] = −0.33, *p* = 0.32), loneliness (*rs* [11] = 0.39, *p* = 0.24), or parent-proxy reported outcome variables: anxiety (*rs* [9] = 0.52, *p* = 0.15), depression (*rs* [9] = 0.38, *p* = 0.31), or HRQoL (*rs* [9] = −0.46, *p* = 0.91). Table 1 outlines the self-reported and proxy ratings of anxiety, depression and HRQoL.

**Figure 2:**
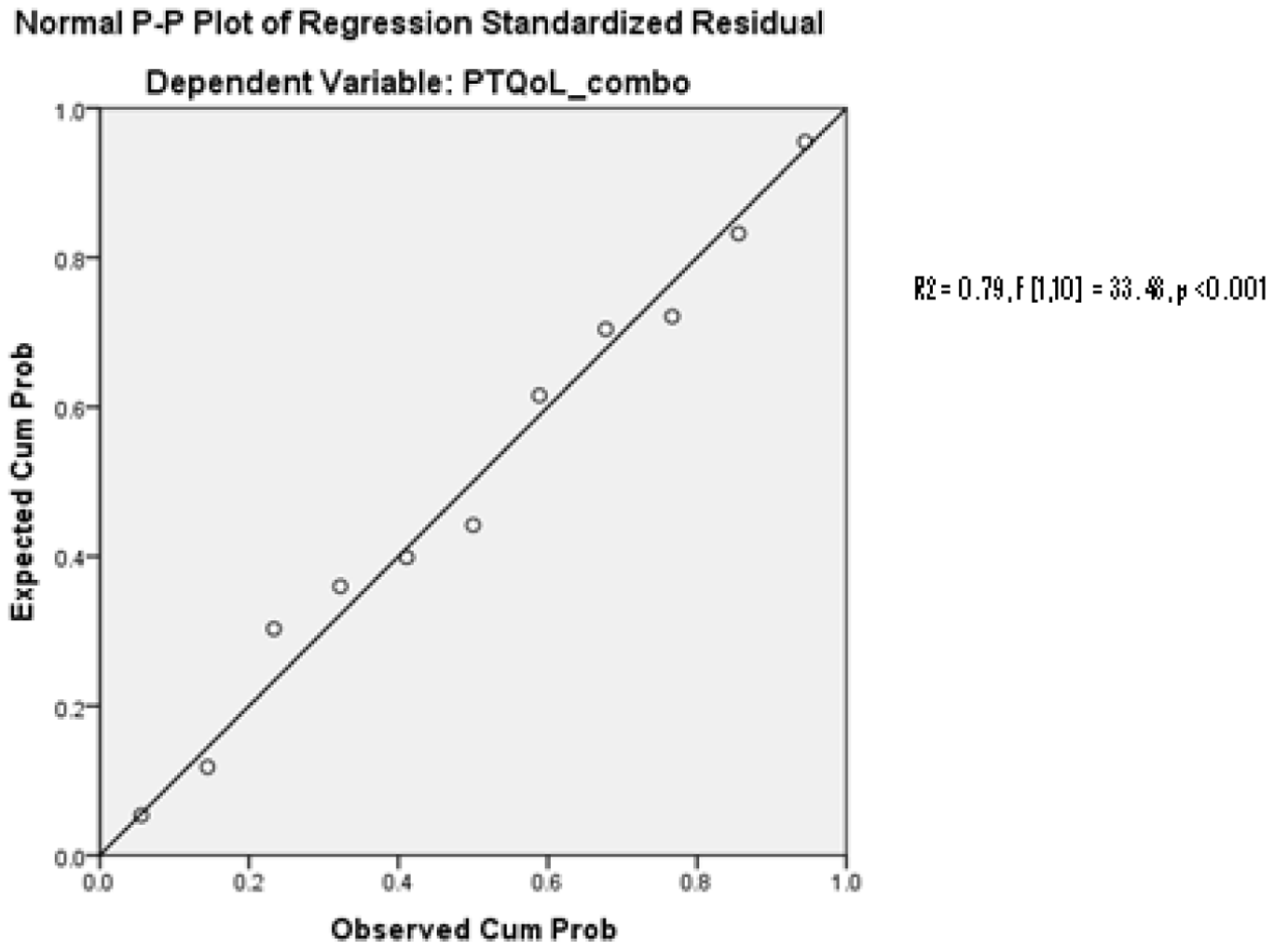
Regression Model, Self-Reported Depression and HRQoL. Cumulative distribution functions (fit between probability distributions) of self-reported depression against self-reported HRQoL. The Probability-Probability (P-P) plot demarks the fit of probability distributions, The data presented are approximately linear, which suggests that the specified theoretical distribution was the correct model (e.g. a good fit between the specific distribution and the observed data).

The regression model with a single predictor (self-reported depression) found that self-reported depression predicted self-reported HRQoL (R^2^ = 0.79, F [1, 10] = 33.48, *p*<0.001) (*see* *Figure 2*). A separate single predictor regression using age at injury found that age at injury was not a significant predictor of self-reported HRQoL (R^2^ = 0.19, F [1, 10] = 2.09, *p* = 0.18).

## Discussion

Our results suggest that self-reported current depressive symptoms predict self-reported current HRQoL in adolescent survivors of a TBI. Importantly, the causality of the relationship, that is, does depression predict quality of life, or does quality of life predict depression, remains unclear at this stage. What is apparent is the significant relationship between the two domains, and that their potentially synergistic association. This seems so for young people for whom their TBI has caused limitations in activities that had been a big part of the adolescents’ life before the injury. For example, one participant expressed grief about no longer being able to ride his bike, which had been an important part of his pre-injury life. There also seemed to be a relationship between what the adolescent had hoped to do in future and an impact on mood, for example, one adolescent was upset at the loss of opportunity to learn how to drive. He was especially sad because his friends were currently meeting this developmental goal without him. While age at injury was correlated with HRQoL, it did not predict HRQoL in our sample. Neither anxiety nor loneliness was associated with HRQoL. As expected, self-report and parent proxy reports were non-concordant.

Hornerman et al. [18] found that at 10 years post-injury, adolescents and young adults experience worse quality of life than healthy controls and those who have undergone organ transplantation when compared on domains of mobility, vision, hearing, eating, speech, mental status, depression, distress and usual activity involvement. When these data are considered against our sample, which were on average 4.6 years post-injury, it could be hypothesized that early identification and treatment of depression could prevent ongoing depression symptoms overtime. Recent adult TBI literature reported an increase in major depressive disorder and generalized anxiety disorder in adults with a severe TBI at 18 months post-injury, which impact on HRQoL [40].

Interestingly we found a relationship between age at injury and self-reported quality of life, where younger age at injury was correlated with a better HRQoL. When a TBI occurs early in childhood, the young person may have little recall of pre-injury life. It is possible that a younger age at injury resulting in a less dramatic life-change for the adolescent, who may have experience life as a continuation post-trauma, versus a change due to the injury. For example, one of the participants stated that he did not know a life pre-injury because he was too young to remember a life prior to the trauma. Severity of injury was not correlated with any of the outcome variables in either parents or self-reports. This result is particularly interesting, given the temptation to assume that mood and quality of life must be impaired as a result of a more significant injury.

While anxiety was not found to be a statistically significant contributor to HRQoL, it was related to both age at testing and loneliness. It is possible that anxiety and loneliness may interact in such a way as to influence the ongoing social development trajectory of these adolescents. Current findings suggest a sequential nature of depressive co-morbidity, e.g. where the onset of depression follows the onset of most anxiety subtypes [41], suggesting that further examination of possible interaction effects of anxiety and depression in adolescent TBI survivors is warranted.

A systematic review found that children as young as 6 years of age were competent in providing reliable, valid accounts of their health [42], the overreliance on parental proxies in TBI research [6] may be due to concerns regarding individual insight into their TBI and less so attributable to the validity of response due to age. Although this study did not assess insight outright, it may be inferred that insight is intact in the adolescent sample assessed in this study, given up to 36% endorsed depression, anxiety, loneliness and quality of life related deficits. Endorsement of these symptoms requires that the individual is aware of their current level of functioning, at an emotional level. While the question regarding insight into the actual TBI is unknown in this sample, the results suggest that insight into one’s own emotional state in a reasonable proportion of the sample is intact. By the same token, the non-significant, inverse relationship between parental proxies and adolescents suggests that the awareness of parents to mood and or quality of life related issues is not objective and may be inaccurate.

## Limitations

The sample size for this study was small, therefore generalizations to all adolescents with a TBI cannot be made and our pilot results must be interpreted with caution. While every attempt was made to encourage participation of adolescents within our reach, unfortunately we were unable to satisfy a large sample. Federal privacy legislation and resultant restrictive methods for identify potential participants (clinical audit) limited our sample size which potentially introduces bias into the sample. The sample may be biased towards unusually keen families, or perhaps those who had personal reasons for participation, such as personal benefit or an opportunity to speak about their experiences. Small sample sizes are a common limitation in TBI outcome research; speculation on effective ways to address this problem may be to involve more interaction between research groups and divisions across the hospital setting, to organize a systematic approach of families, who may be overwhelmed with multiple research requests. Importantly, adolescents are entirely within their rights to decline participation in research studies, regardless of whether or not their parents consent. The failure to achieve a large samples size is partially reflected by adolescents’ authority to say ‘no’ to research studies, which overrides parental interest in research. Efforts to ask adolescents why they do and do not wish to participate in research studies may help to provide insights into how to better market research studies to youth. Sample size limitations must also be considered in light of our attrition analyses, which revealed no significant differences between participants and non-participants on TBI severity, age at injury or gender. The attrition analyses suggest that although our pilot sample was small it was representative of the available pool of adolescents who experienced a TBI on key factors (TBI severity, gender and age). Importantly, small sample sizes do not preclude statistical analyses; rather, they require specific statistical analyses appropriate to small samples. The key limitation of using a small sample size is low power to detect large differences between designs or measures [43]. Of note, the results of the current study identified large effects from a small sample, which suggests that the findings are valid for the sample assessed in this study. Future research on larger samples is required to determine if our findings are generalizable to all survivors of adolescent TBI. Importantly, should depression continue to be a strong predictor of HRQoL in this group, routine assessment of depressive symptoms and HRQoL may help to inform targeted, individual-specific rehabilitation strategies aimed at ameliorating depressive symptomatology and improving HRQoL. Re-assessment of the role of anxiety and loneliness may also be relevant, as a large sample size may yield alternate trends to those reported here. Family history of psychiatric diagnoses were beyond the scope of the current study, but future research may wish to examine what role, if any, family history of psychiatric diagnoses play in adolescent experiences following a TBI. The current study did not collect contextual data regarding participants’, including family situations, which may have impacted on their psychological well-being.

Future research should also consider alternate definitions of quality of life beyond the HRQoL model, especially considering the importance of emotional states described in this study. Assessing the subjective-well being of adolescents may be especially well equipped to disentangle the relationships between mood and QoL.

While our sample size is small and results must be interpreted with caution, this pilot study supports a directional relationship between depression and reduced HRQoL in adolescent survivors of a TBI. Age at injury was correlated with HRQoL, but was not a statistically significant predictor of HRQoL in this sample. Neither loneliness nor anxiety was directly correlated with HRQoL, but they were related to each other. Age at injury was related to HRQoL, but was unable to predict it. As expected, parent proxy and self-reports of anxiety, depression and HRQoL were non-concordant.

Anxiety and depression are the most commonly occurring mental health concerns in otherwise healthy youth. Prevalence and incidence data on anxiety and depression in youth often rely on proxy reporting; despite evidence to suggest that proxy reporting is invalid for this purpose. Anxiety and depression are highly co morbid conditions in the general public. TBI has been consistently linked to new onset or worsening of persisting depression and anxiety across the lifespan, spanning childhood to adulthood. Recent research has supported a link between younger age at injury and development of new onset anxiety disorders, with novel depressive disorders co-morbid with these anxious states. Taken together, there is a reasonable suggestion that adolescent survivors of a TBI are at an increased risk for developing or worsening of anxious and or depressive states, given the impact of sequelae following injury that may interfere with their cognitive, psychosocial and interpersonal functioning. The findings from this pilot study support an important predictive role for depressive symptoms on self-reported HRQoL in adolescent survivors of a TBI.
